# Dynamics of RAS/BRAF Mutations in cfDNA from Metastatic Colorectal Carcinoma Patients Treated with Polychemotherapy and Anti-EGFR Monoclonal Antibodies

**DOI:** 10.3390/cancers14041052

**Published:** 2022-02-18

**Authors:** Anna Maria Rachiglio, Laura Forgione, Raffaella Pasquale, Carlo Antonio Barone, Evaristo Maiello, Lorenzo Antonuzzo, Antonino Cassata, Giuseppe Tonini, Roberto Bordonaro, Gerardo Rosati, Alberto Zaniboni, Sara Lonardi, Daris Ferrari, Giovanni Luca Frassineti, Stefano Tamberi, Salvatore Pisconti, Francesca Di Fabio, Cristin Roma, Armando Orlandi, Tiziana Latiano, Angela Damato, Giampaolo Tortora, Carmine Pinto, Nicola Normanno

**Affiliations:** 1 Cell Biology and Biotherapy Unit, Istituto Nazionale Tumori “Fondazione G. Pascale”-IRCCS, 80131 Naples, Italy; am.rachiglio@istitutotumori.na.it (A.M.R.); l.forgione@istitutotumori.na.it (L.F.); r.pasquale@istitutotumori.na.it (R.P.); c.roma@istitutotumori.na.it (C.R.); 2 Fondazione Policlinico Universitario Agostino Gemelli, 00168 Rome, Italy; carloantonio.barone@policlinicogemelli.it (C.A.B.); armando.orlandi@policlinicogemelli.it (A.O.); giampaolo.tortora@policlinicogemelli.it (G.T.); 3 IRCCS Casa Sollievo della Sofferenza, 71013 San Giovanni Rotondo, Italy; e.maiello@libero.it (E.M.); t.latiano@operapadrepio.it (T.L.); 4 Medical Oncology Unit, Azienda Ospedaliero Universitaria Careggi, 50134 Florence, Italy; lorenzo.antonuzzo@gmail.com; 5 Medical Oncology Unit, Istituto Nazionale Tumori “Fondazione G. Pascale”-IRCCS, 80131 Naples, Italy; a.cassata@istitutotumori.na.it; 6 Medical Oncology Unit, Università Campus Bio-Medico, 00128 Rome, Italy; g.tonini@unicampus.it; 7 Medical Oncology Unit, ARNAS Garibaldi, 95122 Catania, Italy; oncoct@hotmail.com; 8 Medical Oncology Unit, Ospedale San Carlo, 85100 Potenza, Italy; oncogerry@yahoo.it; 9 Fondazione Poliambulanza, 25124 Brescia, Italy; alberto.zaniboni@poliambulanza.it; 10 Medical Oncology IOV, 35128 Padua, Italy; sara.lonardi@iov.veneto.it; 11 Ospedale S. Paolo, 20142 Milan, Italy; daris.ferrari@unimi.it; 12 Medical Oncology Unit, Istituto Scientifico Romagnolo per lo Studio e la Cura dei Tumori (IRST) IRCCS, 47014 Meldola, Italy; luca.frassineti@irst.emr.it; 13 Ospedale per Gli Infermi, 48018 Faenza, Italy; stefano.tamberi@auslromagna.it; 14 Medical Oncology Division, S. Giuseppe Moscati Hospital, 74010 Taranto, Italy; salvatorepisconti@hotmail.it; 15 Medical Oncology Unit, S. Orsola-Malpighi Hospital, 40138 Bologna, Italy; francesca.difabio@aosp.bo.it; 16 Medical Oncology Unit, Clinical Cancer Center, AUSL-IRCCS Reggio Emilia, 42122 Reggio Emilia, Italy; angela.damato@ausl.re.it (A.D.); carmine.pinto@ausl.re.it (C.P.); 17 Department of Medical Biotechnologies, University of Siena, 53100 Siena, Italy

**Keywords:** metastatic colorectal cancer, liquid biopsy, cell-free DNA, anti-EGFR therapy

## Abstract

**Simple Summary:**

Increasing evidence suggests that circulating cell-free DNA (cfDNA) testing might allow for monitoring the response to anti-EGFR monoclonal antibodies in patients with metastatic colorectal carcinoma (mCRC). However, few data are available in treatment-naïve patients. We tested cfDNA samples obtained from mCRC patients enrolled in a phase III trial of the anti-EGFR monoclonal antibody cetuximab plus chemotherapy as first-line treatment. Analysis of serial plasma samples revealed a complex dynamic of RAS/BRAF mutations in response to treatment, with transitory peaks of these mutations that were not associated with resistance to therapy. Overall, our findings suggest that early appearance of RAS/BRAF mutations in the plasma of patients receiving first-line anti-EGFR agents in combination with chemotherapy should not be considered as marker of resistance.

**Abstract:**

Analysis of plasma-derived cell-free DNA (cfDNA) might allow for the early identification of resistance in metastatic colorectal carcinoma (mCRC) patients receiving anti-EGFR monoclonal antibodies. We tested plasma samples from the Erbitux Metastatic Colorectal Cancer Strategy (ERMES) phase III trial of FOLFIRI+Cetuximab in first-line treatment of RAS/BRAF wild-type mCRC. Samples were collected at baseline (*n* = 37), at 8 weeks of treatment (*n* = 32), progressive disease (PD; *n* = 36) and 3 months after PD (*n* = 21). cfDNA testing was performed using the Idylla™ ctKRAS and ctNRAS-BRAF tests and the Oncomine Pan-Cancer Cell-Free Assay. Analysis of basal samples revealed RAS/BRAF mutations in 6/37 cases. A transient RAS positivity not associated with PD was observed at 8 weeks in five cases that showed no mutations at baseline and PD. The frequency of mutant cases increased at PD (33.3%) and decreased again at 3 months after PD (9.5%). The median progression-free survival (mPFS) of patients RAS/BRAF mutant at PD was 7.13 months versus 7.71 months in wild-type patients (*p* = 0.3892). These data confirm that the occurrence of RAS/BRAF mutations in mCRC patients receiving anti-EGFR agents is relatively frequent. However, the cfDNA dynamics of RAS mutations in patients treated with anti-EGFR agents plus polychemotherapy are complex and might not be directly associated with resistance to treatment.

## 1. Introduction

The epidermal growth factor receptor (EGFR) signaling plays a relevant role in the pathogenesis and progression of colorectal carcinoma (CRC) [1,2]. In this respect, the addition of anti-EGFR monoclonal antibodies to first-line polychemotherapy increases the overall response rate (ORR) and prolongs the progression-free survival (PFS) and overall survival (OS), as compared with chemotherapy alone, in metastatic CRC (mCRC) patients who do not carry either KRAS or NRAS mutations [3,4,5]. In fact, the presence of RAS mutations leads to constitutive activation of signaling pathways downstream the EGFR, thus leading to primary resistance to EGFR blockade [6]. The role of BRAF mutations in the resistance to anti-EGFR antibodies is less defined. Although the presence of BRAF mutations does not contraindicate the use of anti-EGFR antibodies, patients with BRAF mutant cancer have a poor prognosis due to the relative resistance of this tumor type to chemotherapy and often receive more aggressive therapeutic regimens [7,8].

In addition to primary resistance mechanisms, the activity of anti-EGFR monoclonal antibodies in CRC is limited by the development of molecular alterations producing acquired resistance [9,10,11,12,13,14,15]. In particular, it has been demonstrated that a fraction of patients with a KRAS/NRAS wild-type CRC before treatment with anti-EGFR antibodies will eventually develop RAS mutations at the progression of the disease. This phenomenon has been confirmed in a number of different reports, although the rate of patients who become RAS mutant at progression significantly differs among the various studies [16,17,18,19,20,21,22,23,24].

Importantly, the mechanisms of acquired resistance to EGFR monoclonal antibodies in mCRC patients were discovered though testing the cell-free DNA (cfDNA) isolated from plasma. The analysis of cfDNA has several advantages over tissue testing, including the possibility to repeat the test over the time, thus monitoring the molecular evolution of the disease.

Studies have shown that the appearance of RAS mutations in cfDNA anticipates clinical progression of the disease [20]. These data have led to the hypothesis that treatment with anti-EGFR drugs should be interrupted when RAS mutations appear, and be restored when the liquid biopsy becomes negative again. However, the majority of data regarding the dynamics of RAS mutations in the cfDNA have been obtained in patients receiving single agent anti-EGFR monoclonal antibodies in advanced lines of treatment. In order to explore the possible role of cfDNA analysis in the first-line treatment of mCRC patients, we tested cfDNA samples obtained from patients enrolled in the Erbitux Metastatic Colorectal Cancer Strategy (ERMES) study. ERMES is a prospective randomized phase III trial of FOLFIRI + Cetuximab until progression (arm A) compared to eight cycles of FOLFIRI + Cetuximab followed by Cetuximab alone until progression (arm B) in first-line treatment of KRAS/NRAS/BRAF wild-type mCRC patients. The study was designed as a non-inferiority trial to investigate whether the PFS in arm B is non-inferior to the PFS in arm A [25].

The availability of plasma samples from patients enrolled in the ERMES trial offers a unique opportunity to study the dynamics of RAS/BRAF mutations in the cfDNA under the pressure of both polychemotherapy and anti-EGFR antibodies, and to evaluate the prognostic and predictive role of cfDNA testing in RAS/BRAF wild-type patients receiving first-line anti-EGFR-based therapy. In particular, we evaluated whether early cfDNA testing might allow for identifying patients with RAS/BRAF mutations, leading to resistance to anti-EGFR agents, who thus might benefit from a different therapeutic strategy.

## 2. Materials and Methods

### 2.1. Patients and Plasma Samples

The ERMES study is a phase III randomized trial of FOLFIRI+Cetuximab until progressive disease (PD, arm A) compared to 8 cycles of FOLFIRI+Cetuximab followed by Cetuximab alone until PD (arm B) in first-line treatment of KRAS/NRAS/BRAF wild-type mCRC patients. Plasma samples were collected from 37 mCRC patients enrolled in ERMES at baseline (*n* = 37), 8 weeks of treatment (*n* = 32), PD (*n* = 36) and at 3 months after PD (*n* = 21). Overall, 10 patients were randomized in arm A and 27 in arm B.

RAS/BRAF testing in the ERMES trial was performed by using standard of care techniques at peripheral laboratories of participating centers. All patients included in this study were microsatellite stable (MSS) as assessed by local pathology laboratories.

The plasma samples were isolated from 10.0 mL of whole blood in EDTA Vacutainer tubes. After the collection, the blood samples were immediately processed. Cells were removed by centrifugation for 10 min at 1600× *g* using a refrigerated centrifuge and, without disturbing the bottom red blood cell layer, the supernatant from the top layer of the tube was transferred into the new collection tube. Another centrifugation was performed for 10 min at 3000× *g* in order to remove the platelets. The supernatant was transferred in criovials and stored at −80 °C. The plasma samples were shipped at the centralized laboratory in dry ice.

### 2.2. Idylla Analysis

Plasma samples were analyzed using the fully automated Idylla™ ctKRAS and Idylla™ ctNRAS-BRAF mutation test (Biocartis, Mechelen, Belgium). For each sample, 1 mL of plasma was loaded into the Idylla™ cartridge. The Idylla™ ctKRAS test covers 21 KRAS mutations in exons 2, 3 and 4; the ctNRAS-BRAF test covers 18 mutations in exons 2, 3 and 4 of NRAS gene and 5 mutations in BRAF codon 600.

### 2.3. NGS of Plasma Samples

The circulating total nucleic acids (cTNA) were extracted from 4 mL of plasma samples using MagMAX™ Cell-Free Total Nucleic Acid Isolation Kit (Thermo Fisher Scientific, San Diego, CA, USA) and quantified using the Qubit dsDNA HS Assay Kit (Thermo Fisher Scientific). An amount of 2–20 ng of cfTNA was used to prepare libraries. Targeted libraries were performed using the Oncomine Pan-Cancer Cell-Free Assay (Thermo Fisher Scientific), following the manufacturer’s recommendations. The Oncomine Pan-Cancer Cell-Free Assay assesses genetic alterations in 52 driver genes and includes the following: hotspot genes and short indels in AKT1, ALK, AR, ARAF, BRAF, CHEK2, CTNNB1, DDR2,EGFR, ERBB2, ERBB3, ESR1, FGFR1, FGFR2, FGFR3, FGFR4, FLT3, GNA11, GNAQ, GNAS, HRAS, IDH1, IDH2, KIT, KRAS, MAP2K1, MAP2K2, MET, MTOR, NRAS, NTRK1, NTRK3, PDGFRA, PIK3CA, RAF1, RET, ROS1, SF3B1, SMAD4 and SMO; gene fusions in ALK, BRAF, ERG, ETV1, FGFR1, FGFR2, FGFR3, MET, NTRK1, NTRK3, RET and ROS1; MET exon 14 skipping; copy number variations of CCND1, CCND2, CCND3, CDK4, CDK6, EGFR, ERBB2, FGFR1, FGFR2, FGFR3, MET and MYC; and coverage of tumor suppressor genes APC, FBXW7, PTEN and TP53. The final concentration of each library was determined by Ion Library TaqMan™ Quantitation Kit (Thermo Fisher Scientific). Barcoded libraries were diluted to 100 pM, pooled in equal volume aliquots and then loaded on to the Ion Chef™ Instrument (Thermo Fisher Scientific) for emulsion PCR, enrichment and loading onto the Ion S5 540 chip. The sequencing runs were performed on the Ion S5 XL System (Thermo Fisher Scientific). The data were analyzed by Ion Torrent Suite Software v.5.12 and using Ion Reporter Software v5.14.

### 2.4. Targeted Sequencing Analysis of Tumor Tissue

Tissue samples were analyzed with the Oncomine Solid Tumor DNA kit (Thermo Fisher Scientific). The panel analyzes hotspot and targeted regions of the following 22 genes implicated in colon and lung cancers: ALK, EGFR, ERBB2, ERBB4, FGFR1, FGFR2, FGFR3, MET, DDR2, KRAS, PIK3CA, BRAF, AKT1, PTEN, NRAS, MAP2K1, STK11, NOTCH1, CTNNB1, SMAD4, FBXW7 and TP53. Libraries were prepared starting from 10 ng of genomic DNA (measured using the Qubit fluorometer in combination with the Qubit dsDNA HS Assay Kit) according to the manufacturer’s instructions. Then, 100 pM of each equalized library was multiplexed and clonally amplified on Ion sphere particles (ISPs) by emulsion PCR performed on the Ion One Touch 2 instrument with the Ion PGM template OT2 200 kit (Thermo Fisher Scientific). The template ISPs were enriched, loaded on an Ion 316 chip and sequenced on a PGM sequencer with the Ion PGM™ sequencing 200 kit v2 according to the manufacturer’s instructions. The data were analyzed by Ion Torrent Suite Software v.5.12 and using Ion Reporter Software v5.14.

## 3. Results

We analyzed plasma samples obtained from 37 KRAS/NRAS/BRAF wild-type mCRC patients (10 in arm A and 27 in arm B) enrolled in the ERMES trial using the Idylla platform. In particular, we studiedd available plasma samples at baseline (*n* = 37), at 8 weeks of treatment (*n* = 32), at progressive disease (PD; *n* = 36) and at 3 months after PD (*n* = 21). The analysis performed with the Idylla™ ctKRAS/NRAS/BRAF assay was successful for all but one sample at 3 months after PD for whom the KRAS test was invalid (Table 1).

### 3.1. KRAS/NRAS/BRAF cfDNA Status at Baseline

The analysis of the cfDNA isolated form the baseline plasma samples revealed the presence of KRAS mutations in four cases, and of NRAS and BRAF mutations in one case for each gene, with an overall concordance with tissue testing of 83.8% (Table 1).

Targeted sequencing analysis of four available tumor tissue samples from discordant cases showed the presence of the same RAS variant identified in plasma in 3/4 patients, at allelic frequencies ranging between 2.1% and 13.4%.

Plasma samples at 8 weeks and at PD were available for all patients with baseline KRAS/NRAS/BRAF mutations in the cfDNA (Table 1). Patient 29-0001 carried a BRAF V600E variant on liquid biopsy and had a rapid PD (PFS 1.38 months) with the liquid biopsy still positive for BRAF. In this patient, the sample at week 8 coincided with PD. Two RAS-positive cases became negative at week 8 and remained negative at PD (01-0010 and 73-001), while an additional two were negative at week 8 but at PD showed the same RAS mutation that was identified at baseline (01-0011 and 01-0014). Only one patient had a persistent RAS mutation at 8 weeks and at PD (01-0019).

**Figure 1:**
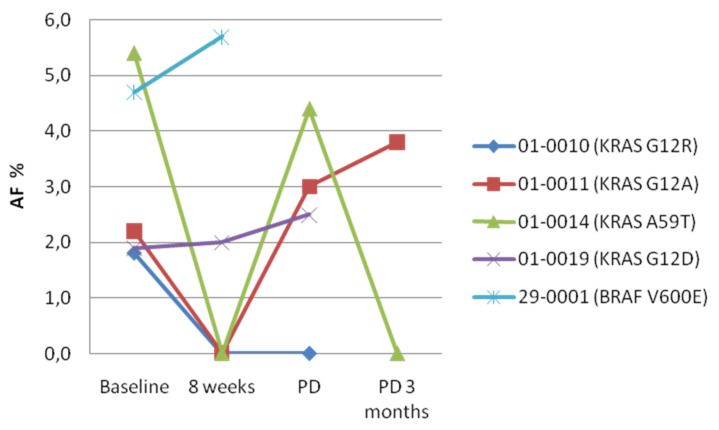
Dynamics of KRAS/NRAS/BRAF mutations in the subgroup of patients with baseline positive cfDNA. Mutant allelic frequency (AF) was assessed by targeted sequencing at the indicated time points. Patient 29-0001 dropped out of the study at week 8 due to early disease progression.

We tested the cfDNA samples of five out of six patients who were positive at baseline with the Oncomine Pan Cancer Cell-Free Assay, which assesses genetic alterations in 52 driver genes, in order to confirm the variants identified by the Idylla™ test, to estimate the allelic frequency and to evaluate the presence of additional variants. cfRNA was assessed as well to identify gene fusions. All the KRAS/NRAS/BRAF variants identified with Idylla™ were confirmed by the Oncomine assay, which did not identify additional variants at baseline (Supplementary Table S1). The dynamics of KRAS/NRAS/BRAF mutations in the subgroup of patients with a baseline positive cfDNA test is shown in Figure 1. The analysis of cfRNA did not reveal the presence of alterations in any sample tested.

### 3.2. KRAS/NRAS/BRAF cfDNA Status at Week 8

Among the 32 available plasma samples obtained at 8 weeks, the test revealed four KRAS, two NRAS and two BRAF mutations in seven patients (22.6% of the available cases). Cases 01-0019 and 29-001 were positive at baseline and at every available subsequent time point (Table 1). Four cases that were positive at 8 weeks had no mutations detectable at baseline and no mutation was detectable at PD. Finally, case 01-0017 had no mutations at baseline, showed a KRAS G12A variant at 8 weeks and was found to carry a BRAF V600 mutations at PD. Targeted sequencing of cfTNA from the seven patients positive at week 8 showed the same genomic alterations identified by the Idylla test (Supplementary Table S1) and did not reveal the presence of gene fusions.

**Figure 2:**
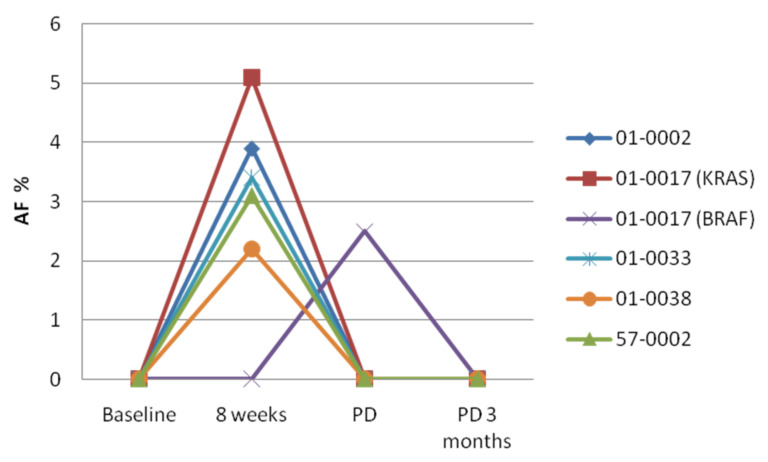
Dynamics of KRAS/NRAS/BRAF mutations in the subgroup of patients with positive cfDNA at 8 weeks. Mutant allelic frequency (AF) was assessed by targeted sequencing at the indicated time points. Patient 01-0017 showed two different variants at week 8 (KRAS G12A) and at PD (BRAF V600).

The peculiar dynamics of these cases with a wave of transient KRAS mutations is shown in Figure 2.

### 3.3. KRAS/NRAS/BRAF cfDNA Status at PD

The fraction of KRAS/NRAS/BRAF cfDNA-positive cases significantly increased at PD, with 14 mutations detected in 12/36 patients (33.3%). In particular, the Idylla™ test identified six KRAS, three NRAS and five BRAF variants, with two cases carrying either a KRAS or NRAS mutation and a BRAF V600E variant (Table 1). An additional patient (29-001) had a very early PD at 8 weeks as above described, and it should be considered a refractory case. Three cases showed at progression the same KRAS mutation identified at baseline, while only one case positive at week 8 showed the same KRAS mutation at baseline and progression (Figure 1 and Figure 2). The results of the Idylla test were confirmed in 20 cases with additional plasma samples available by targeted sequencing, which identified the same KRAS, NRAS and BRAF variants found by Idylla (Supplementary Table S1). The NGS test also revealed three TP53 mutations, two of which found also in the baseline cfDNA sample from the same patients (Supplementary Table S1). In addition, a pathogenic FBXW7 mutation was detected at PD in patient 01-0010, who also carried a KRAS mutation at baseline but not at the other time points. Additionally, at this time point, the analysis of cfRNA did not show the presence of gene fusions.

### 3.4. KRAS/NRAS/BRAF cfDNA Status 3 Months after PD

We had a significant dropout of cases at 3 months after PD. In fact, only 21 patients had available plasma samples at PD. Such dropout is probably due to the fact that patients with disease progression left the study to receive a second line of therapy in the context of clinical practice. In particular, all patients received a second-line regimen not containing an anti-EGFR agent.

Only 2 of the 21 cases with available samples at 3 months after PD (9.5%) showed either a KRAS or a KRAS and NRAS variants (Table 1). Such reduction in the frequency of RAS-positive cases is in agreement with previous studies demonstrating that the interruption of the treatment with anti-EGFR monoclonal antibodies leads to a reduction in the levels of RAS mutations in patients who developed such mutations on therapy [20]. NGS testing confirmed the two KRAS mutations but not the NRAS variant (Supplementary Table S1). In patient 01-0011, targeted sequencing also revealed a TP53 mutation in addition to the KRAS variant, while a EGFR S492R mutation was detected in the cfDNA from patient 01-0014. The analysis of cfRNA did not reveal the presence of alterations.

### 3.5. Correlation between KRAS/NRAS/BRAF cfDNA Status and Patients’ Outcomes

We next assessed the correlation between the presence of KRAS/NRAS/BRAF mutations in the cfDNA and the outcome of patients who received first-line chemotherapy plus cetuximab. The presence of KRAS/NRAS/BRAF mutations at baseline, at 8 weeks, at PD or at any of these points did not correlate with response to therapy (data not shown). However, the dynamics of RAS mutations did correlate with patients’ outcomes on treatment. We had paired 8-week samples available for six cases with RAS or BRAF mutations at baseline. In 4/6 cases, we observed a partial response to therapy that was associated with plasma samples becoming negative at week 8. The only baseline KRAS-positive patient with persistent KRAS mutation at week 8 did not respond to therapy but had a stabilization of the disease. The BRAF-positive case had a PD with a very short PFS of 1.38 months, while showing an increase of the BRAF MAF in the cfDNA (Figure 1).

Interestingly, the four cases with transient RAS mutation increase at week 8 all had PR as the best response, while case 01-0017 with a RAS-positive sample at week 8 and BRAF positivity at PD had CR as the best response.

**Figure 3:**
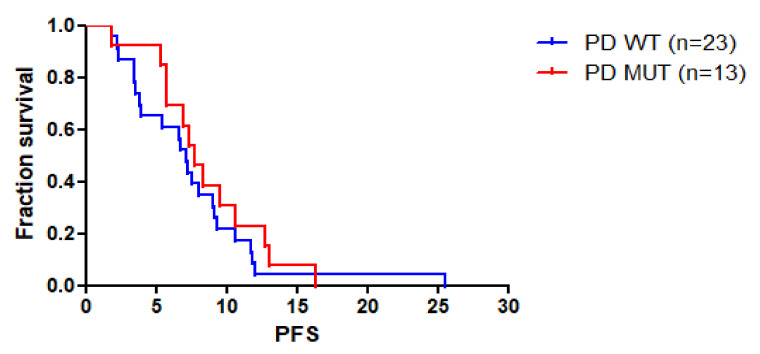
Progression-free survival curves of patients with KRAS/NRAS/BRAF mutations at PD (PD MUT) versus patients with wild-type cases for the three genes at PD (PD WT).

The median progression-free survival (mPFS) of patients with KRAS/NRAS/BRAF mutations at PD was 7.13 months versus 7.71 months in patients with wild-type plasma samples (*p* = 0.3892; HR 1.346, CI 95% 0.6844 to 2.647) (Figure 3). Similarly, no significant difference in mPFS was observed between KRAS/NRAS/BRAF mutant cases versus wild-type cases when considering patients’ mutant status at baseline, at week 8 or at any time-point (Supplementary Table S2).

## 4. Discussion

Testing plasma-derived cfDNA has several advantages over tissue testing, namely being able to better recapitulate the heterogeneity of advanced cancers and allowing for the monitoring of the molecular evolution of the disease [26]. In particular, liquid biopsy testing might provide important information on the response to therapy, thus consenting to adapt the therapeutic strategy. Nevertheless, the clinical interpretation of the results of liquid biopsy testing is often complex and must take into account a number of biological, technical and clinical factors.

Several studies demonstrated a good concordance between tissue and cfDNA testing of RAS mutations in patients with mCRC [24,27,28,29,30,31,32,33]. However, patients with a RAS-positive liquid biopsy but a negative tissue RAS test have been described in different studies [32,34]. Analysis of tumor tissue with highly sensitive techniques confirmed the presence of RAS mutations in the tumor tissue of most of these discordant cases [32,34]. In this respect, we could identify in the tumor tissue the same RAS variant detected in the cfDNA in 3/4 cases classified as RAS wild type based on previous local tissue testing. In addition, the variants identified in plasma with the Idylla™ test were all confirmed by NGS, thus excluding potential artifacts. Therefore, our findings confirm that a fraction of mCRC patients carries sub-clonal RAS/BRAF variants that might not be identified by testing a biopsy of a single tumor site and that can be identified by cfDNA testing [32,35,36,37].

Although RAS/BRAF mutations are well-defined mechanisms of resistance to anti-EGFR agents [38,39,40,41,42,43], the presence of a RAS/BRAF mutation at baseline did not affect the probability to respond to EGFR monoclonal antibodies-based polychemotherapy nor was it associated with shorter PFS in this study. While these data must be cautiously interpreted due to the low number of liquid biopsy baseline RAS/BRAF-positive cases; they also highlight that the predictive role of RAS/BRAF mutations detected in liquid biopsy in patients receiving anti-EGFR agents plus polychemotherapy might be different as compared with anti-EGFR monoclonal antibodies used as single agent in later lines of treatment. For example, the four patients with baseline RAS mutations in the liquid biopsy who experienced PR had a significant decrease in the levels of RAS-positive ctDNA by week 8, suggesting that the RAS mutant clones were sensitive to polychemotherapy. We might still expect that patients with a RAS mutation in the baseline liquid biopsy will have a shorter PFS as compared with RAS wild-type patients, because they will not benefit of the addiction of anti-EGFR agents to chemotherapy. However, patients with RAS/BRAF wild-type tissue and mutant liquid biopsy are likely to carry sub-clonal RAS/BRAF variants, which might only in part affect the efficacy of anti-EGFR drugs [37]. The only patient of the baseline-positive cases who had a PD carried a BRAF V600E variant and had no adequate tissue specimen available for testing. In most cases, the baseline RAS/BRAF mutation was also detected at PD, suggesting that these variants might contribute to tumor progression, but also that additional genomic events are required to drive resistance to both anti-EGFR agents and chemotherapy. Prospective clinical trials are definitely required to define the best therapeutic strategy in patients with baseline sub-clonal RAS/BRAF mutations.

We also observed a transient increase in the levels of RAS mutations at week 8 in five patients who became RAS negative at PD. A transient increase of RAS-positive clones has been previously described in mCRC patients receiving anti-EGFR monoclonal antibodies, who still became RAS positive at PD [23]. It must be emphasized that, in this previous study, the patients did not receive polychemotherapy. We might hypothesize that in our series chemotherapy might have been effective in eradicating RAS-positive sub-clones that were initially selected by anti-EGFR therapy. Importantly, 3/5 of these patients had a PR at week 8, with 2/5 patients showing SD. The best response in this subgroup was PR in three patients, CR in one patient and SD in one patient, while the PFS ranged between 5.38 and 16.34 months. Interestingly, previous studies showed that early increase in RAS mutations in cfDNA in patients treated with anti-EGFR agents is not associated with resistance to treatment [34]. Taken together, these findings argue against the use of liquid biopsy testing as early marker of resistance in mCRC patients receiving anti-EGFR antibodies plus polychemotherapy. Additional studies in larger cohorts of patients are definitely needed to define the true frequency of the phenomenon that we observed.

The rate of RAS/BRAF-positive cases at PD following treatment with an anti-EGFR monoclonal antibody ranged between 32% and 96% in previous studies, a significant difference that was most likely due to the limited number of patients and the different technologies used for testing [16,17,18,19,20,21,22,23,24]. A previous study that employed the Idylla™ test reported RAS/BRAF mutations at PD in 14% of mCRC patients receiving first-line EGFR antibodies-based therapy [18]. In this respect, the 35.1% RAS-positive rate at PD observed in our study is significantly higher. Such difference could be due to a number of issues, including the fact that the blood draws in our trial was performed at PD, whereas in the study by Maurel and co-workers, blood was drawn within 1 month before PD or after PD, thus possibly limiting the sensitivity of the assay. Importantly, in both studies the emergence of RAS/BRAF mutations did not correlate with PFS, suggesting that the presence of these variants does not correlate with a more aggressive phenotype. However, we must acknowledge that the true positive fraction of cases could be underestimated because of the limited sensitivity of the tests that we used. In this respect, the estimation of tumor fraction in plasma samples might in the future allow for a better definition of this phenomenon [44,45,46]. Studies on larger patients’ cohorts will allow us to better define the prognostic value of acquired, likely sub-clonal RAS and BRAF variants, which could be different as compared with de novo clonal mutations of these genes.

## 5. Conclusions

This study has several limitations due to the low number of patients included in our analysis. However, our preliminary findings suggest a complex dynamic of RAS/BRAF mutations in mCRC patients treated with first-line anti-EGFR monoclonal antibodies in combination with polychemotherapy. Additional studies are needed to clear whether cfDNA monitoring might guide therapeutic decisions in mCRC patients.

## Figures and Tables

**Table 1:** Summary of cfDNA results by Idylla^TM^ ctKRAS/NRAS/BRAF assays.

ID	ARM	Baseline	8 Weeks	PD	PD 3 Months
**01-0001**	B	–	–	–	–
**01-0002**	B	–	**KRAS**: p.G12S; **BRAF**: p.V600E/D	–	–
**01-0005**	A	–	–	**NRAS**: p.Q61R/K	–
**01-0007**	A	–	–	–	–
**01-0008**	B	–	–	**KRAS**: p.G12C	NA
**01-0010**	A	**KRAS**: p.G12R	–	–	NA
**01-0011**	B	**KRAS**: p.G12A	–	**KRAS**: p.G12A	**KRAS**: p.G12A
**01-0012**	B	–	–	–	NA
**01-0013**	B	–	–	–	NA
**01-0014**	B	**KRAS**: p.A59T/E/G	–	**KRAS**: p.A59T/E/G	Invalid
**01-0015**	A	–	NA	–	NA
**01-0017**	B	–	**KRAS**: p.G12A	**BRAF**: p.V600E/D	–
**01-0019**	A	**KRAS**: p.G12D	**KRAS**: p.G12D	**KRAS** p.G12D	NA
**01-0024**	B	–	–	**NRAS**: p.Q61H	–
**01-0027**	B	–	–	**KRAS**: p.G12R	–
**01-0028**	A	–	–	–	**KRAS**: p.G12V; **NRAS**: p.Q61H
**01-0030**	B	–	–	–	–
**01-0031**	B	–	NA	–	–
**01-0032**	B	–	–	–	NA
**01-0033**	B	–	**NRAS**: p.A59T	–	–
**01-0036**	A	–	NA	–	–
**01-0038**	B	–	**NRAS**: p.G12A/V	–	–
**61-0001**	B	–	NA	**BRAF**: p.V600E/D	NA
**61-0003**	A	–	–	–	–
**06-0002**	B	–	NA	**KRAS**: p.A146P/T/V; **BRAF**: p.V600E/D	NA
**29-0001**	B	**BRAF**: p.V600E/D	**BRAF**: p.V600E/D	*	NA
**26-0002**	B	–	–	**BRAF**: p.V600E/D	–
**33-0004**	B	–	–	**NRAS**: p.Q61R/K; **BRAF**: p.V600E/D	NA
**63-0006**	B	–	–	–	NA
**73-0001**	B	**NRAS**: p.Q61H	–	–	NA
**57-0002**	A	–	**KRAS**: p.A146P/T/V	–	–
**57-0003**	A	–	–	–	–
**57-0005**	B	–	–	–	NA
**57-0006**	B	–	–	**KRAS**: p.G12C	–
**19-0006**	B	–	–	–	NA
**65-0004**	B	–	–	–	NA
**70-0001**	B	–	–	–	–

NA: sample not available; *: the sample at week 8 coincided with PD.

## Data Availability

Publicly available datasets were analyzed in this study. This data can be found here: https://data.mendeley.com/datasets/cng6gnp8k8/draft?a=8d191a3b-d52b-4f39-8967-a03d0480d47a (accessed on 11 November 2021).

## Supplementary Materials

The following supporting information can be downloaded at: https://www.mdpi.com/article/10.3390/cancers14041052/s1, Table S1: Comparison of Idylla^TM^ and Oncomine Pan Cancer results, Table S2: mPFS observed between KRAS/NRAS/BRAF mutant versus wild-type cases when considering patients’ mutant status at baseline, at week 8, at progression or at any time point.
