# Supplementary material for: Dynamics of RAS/BRAF Mutations in cfDNA from Metastatic Colorectal Carcinoma Patients Treated with Polychemotherapy and Anti-EGFR Monoclonal Antibodies
Source: Cancers (Basel). 2022 Feb 18;14(4):1052. doi: 10.3390/cancers14041052 (PMC8870112; doi:10.3390/cancers14041052)
Supplement: Supplementary file 1 [file cancers-14-01052-s001.zip › cancers-1551702-supplementary.pdf]

Table S1: Comparison of Idylla and Oncomine Pan Cancer results.

ID	Baseline		8 Weeks		PD		PD 3 Months	
	Idylla	NGS (AF%)	Idylla	NGS	Idylla	NGS	Idylla	NGS
**01-0002**	–	–	**KRAS:** p.G1[illegible] **BRAF:** p.V600E/D	**KRAS:** p.G12S (3,9%); **BRAF:** p.V600D (1,8%)	–	–	–	–
**01-0005**	–	**TP53:** p.R273C (14,6%)	–	**TP53:** p.R273C (11,2%)	**NRAS:** p.Q61R/K	**NRAS:** p.Q61K (2,8%); **TP53:** p.R273C (7,8%)	–	–
**01-0008**	–	–	–	–	**KRAS:** p.G12C	**KRAS:** p.G12C (6,4%)	sample not available	
**01-0010**	**KRAS:** p.G12R	**KRAS:** p.G12R (1,8%)	–	–	–	**FBXW7:** p.R465C (1,5%)	sample not available	
**01-0011**	**KRAS:** p.G12A	**KRAS:** p.G12A (2,2%)	–	–	**KRAS:** p.G12A	**KRAS:** p.G12A (3%); **TP53:** p.R280I (2,5%)	**KRAS:** p.G12A	**KRAS:** p.G12A (3,8%); **TP53:** p.R280I (2,9%)
**01-0014**	**KRAS:** p.A59T/E/G	**KRAS:** p.A59T (5,4%)	–	–	**KRAS:** p.A59T/E/G	**KRAS:** p.A59T (4,4%)	Invalid	**EGFR:** p.S492R (0,4%)
**01-0017**	–	–	**KRAS:** p.G12A	**KRAS:** p.G12A (5,1%)	**BRAF:** p.V600E/D	**BRAF:** p.V600E (2,5%)	–	–
**01-0019**	**KRAS:** p.G12D	**KRAS:** p.G12D (1,9%)	**KRAS**: p.G12D	**KRAS**: p.G12D (2%)	**KRAS:** p.G12D	**KRAS:** p.G12D (2,5%)	sample not available	
**01-0024**	–	–	–	–	**NRAS:** p.Q61H	**NRAS:** p.Q61H (4,1%)	–	–
**01-0027**	–	–	–	–	**KRAS:** p.G12R	**KRAS:** p.G12R (3,4%)	–	–
**01-0028**	–	–	–	–	–	–	**KRAS:** p.G12V; **NRAS:** p.Q61H	**KRAS:** p.G12V (2,4%)
**01-0033**	–	–	**NRAS:** p.A59T	**NRAS:** p.A59T (3,4%)	–	–	–	–
**01-0038**	–	–	**NRAS:** p.G12A/V	**NRAS:** p.G12V (2,2%)	–	–	–	–
**61-0001**	–	–	sample not available		**BRAF:** p.V600E/D	**BRAF:** p.V600E (1,9%)	sample not available	
**06-0002**	–	**TP53:** p.R175H (5,4%)	sample not available		**KRAS:** p.A146P/T/V; **BRAF:** p.V600E/D	**KRAS:** p.A146T (1,5%); **BRAF:** p.V600E (3,2%); **TP53:** p.R175H (2,8%)	sample not available	
**29-0001**	**BRAF:** p.V600E/D	**BRAF:** p.V600E (4,7%)	**BRAF**: p.V600E/D	**BRAF:** p.V600E (5,7%)	the sample at week 8 coincided with PD		sample not available	
**26-0002**	–	–	–	–	**BRAF:** p.V600E/D	**BRAF:** p.V600E (2,6%)	–	–
**33-0004**	–	–	–	–	**NRAS:** p.Q61R/K; **BRAF:**p.V600E/D	**NRAS:** p.Q61K (4,8%); **BRAF:** p.V600D (2,6%)	sample not available	
**57-0002**	–	–	**KRAS:** p.A146P/T/V	**KRAS:** p.A146T (3,1%)	–	–	–	–
**57-0006**	–	–	–	–	**KRAS:** p.G12C	**KRAS:** p.G12C (4,3%)	–	–

Table S2. mPFS observed between KRAS/NRAS/BRAF mutant versus wild type cases when considering patients mutant at baseline, at week 8, at progression or at any time-point.

**Samples**		**N**	**MEDIAN PFS**	***P* Value**	**Hazard Ratio (95% CI of Ratio)**
**T0**	**WT**	31	7.250	0.5818	0.7625 (0.2904 to 2.002)
	**MUT**	6	7.060		
**T8**	**WT**	25	7.590	0.8401	1.090 (0.4724 to 2.514)
	**MUT**	7	6.670		
**PD**	**WT**	23	7.130	0.3892	1.346 (0.6844 to 2.647)
	**MUT**	13	7.710		
**Any Time Point**	**WT**	17	7.130	0.5476	1.230 (0.6269 to 2.412)
	**MUT**	20	7.470		
